# Sickle Cell Trait and Sudden Death

**DOI:** 10.1186/s40798-018-0131-6

**Published:** 2018-05-23

**Authors:** Bruce L. Mitchell

**Affiliations:** Emory University Hospital Midtown, 550 Peachtree Street, NE, Atlanta, Ga 30308 USA

**Keywords:** Sickle cell trait, Sudden death, Rhabdomyolysis, Exertional, Sickling

## Abstract

Sickle cell trait has long been considered a benign condition but continues to be the leading cause of death in young African Americans in military basic training and civilian organized sports. There continues to be a great deal of controversy surrounding sickle cell trait and its association with exercise-related morbidity and sudden death. Even though sickle cell trait has a high prevalence among African Americans, many clinicians believe the potential adverse consequences should have been mitigated by actions such as universal screening in the USA at birth for sickle hemoglobin, National Collegiate Athletic Association (NCAA) rule changes, and changes in the US Military boot camp system. Sudden death due to periods of extreme physical exertion continues to occur in individuals with sickle cell trait.

## Key Points

1. Sickle cell trait is not a benign condition and continues to be associated with sudden death in individuals during periods of extreme physical exertion.
2. While many factors likely contribute to exertional collapse in individuals with sickle cell trait, the exact mechanism has not been fully elucidated.
3. Multiple key stakeholders have agreed to label the clinical presentation as “exercise collapse associated with sickle cell trait.”

## Review

There is still a great deal of controversy surrounding sickle cell trait and its association with exertional collapse and sudden death. Although most individuals have little or no clinical sequelae, debate centers on the effects of extreme physical exertion. The large number of African Americans that serve in the US Military and those that participate in endurance athletics are particularly at risk. This article reviews the epidemiology and pathophysiology of sickle cell trait and describes the risks and complex interactions of multiple physiologic factors associated with exertional collapse, as well as screening and management of those at risk.

The syndrome has the highest prevalence among individuals of African descent but is also exhibited in those of Caribbean, Arab, East Indian, and Mediterranean lineage. While the author recognizes race as a social construct, the terms “African American” (AA) and “black” are used interchangeably to denote individuals of African lineage who are likely citizens of the USA (AA) and identify phenotypically as black vs “black” individuals who may not be citizens of the USA but still are recognized as being of African lineage.

### Epidemiology

Sickle syndromes include several distinct diseases that cause red blood cells to sickle in vivo. The most recognized are sickle cell anemia, sickle cell trait, hemoglobin sickle cell disease, and sickle cell-β-thalassemia. In the USA, of all the hemoglobinopathies, individuals with homozygous sickle genes (HgbSS) have the greatest morbidity and mortality, and the disease limits their ability to participate in most organized athletic activities. Sickle cell trait is the heterozygous condition (HgbAS) and has a prevalence rate of 6 to 9% in African Americans and 0.01 to 0.05% of the remaining population primarily those with East Indian, Hispanic, Arab, and Mediterranean lineage [1–3].

### Pathophysiology

The normal hemoglobin molecule has four subunits each carrying an oxygen-laden heme group and a globin molecule. In adult hemoglobin, two alpha subunits are bound to two β-subunits (HBA, α2β2). Sickle hemoglobin (HgbS) occurs when a hydrophobic valine replaces a hydrophilic glutamic acid creating two mutant β-subunits which combine with two normal α-subunits.

When sickle hemoglobin is deoxygenated, the molecules have an increased tendency to form hydrophobic bonds and will subsequently aggregate into large polymers on the red blood cell membrane. The rate and extent of polymer formation are dependent on four factors: intracellular hemoglobin concentration, level of pH, presence or absence of hemoglobin F, and the degree of oxygenation in the cell. In sickle cell trait, the hemoglobin concentration is normal, and hemoglobin F is not usually present postnatally. Therefore, it is postulated that the predominant physiologic cause of intravascular sickling or rheological impairment in those with heterozygous sickle genes (HgbAS) is the low level of oxygenation in the cell and a decreasing pH (acidosis) which in a positive feedback loop then trigger skeletal muscle metabolic failure or rhabdomyolysis [3].

It is well known that HgbAS-containing red blood cells can be induced to sickle in vitro within minutes [4, 5]. US Army research has shown that intravascular sickling can occur in minutes just by doing maximal arm cranking exercises [6]. Clinical and pathological evaluation of several individuals who survived long enough for medical intervention determined that all had exerted effort beyond their conditioning level and all presented with metabolic acidosis soon followed by rhabdomyolysis, renal failure, and disseminated intravascular coagulation [7, 8].

There is likely some contribution of the different types of muscle fibers found in different proportions based on genotype [9, 10]. Black African men had significantly more type IIa fibers (fast twitch 49 vs 42%) and fewer type I fibers (slow twitch 33 vs 41%) than Caucasian men [10]. Also, the muscle activities of several glycolytic pathway enzymes were 40 to 76% higher in black than Caucasian men and the average creatine kinase levels twice as high [10]. Blood viscosity and adhesion factors are altered in individuals with sickle cell trait [10, 11].

Other genetic factors may also play a role in the increased susceptibility of blacks to exertional sickling. The rapid rise in cytoplasmic calcium is necessary for muscle contraction and is the result of calcium release through type I ryanodine receptors (RYR1) [12]. Analysis of RYR1 receptors is used as a screening test for susceptibility to malignant hyperthermia, and some have suggested a relationship between RYR1 receptor variants, Exercise Heat Illness (EHI), and exertional rhabdomyolysis in those with sickle cell trait [13].

Even though the majority of data in the sickle syndrome literature [7, 14–17] showed that sickle cell trait was associated with increased risk of exercise-induced sudden death (independent of pre-existing disease), the inability to discern histologically artefactual postmortem sickling from anti-mortem sickling has left the association open to debate. Even though causal relationship is plausible, no direct evidence links the pathogenesis of exercise-related death to microvascular obstruction by rigid erythrocytes.

### Military Deaths

The first cases identified were four US Army recruits who died during or immediately after strenuous exercise during basic training at Fort Bliss, Texas (elevation 4050 ft.), between March 1968 and February 1969 [18]. On the basis of autopsy specimens and morphologic criteria previously established for individuals with sickle cell disease [19, 20], the authors concluded that the soldiers died of diffuse microvascular obstruction from sickled erythrocytes or “sickle crisis.” Although hypoxemia was modest at 4000 ft (ambient PO2, 70–75 mmHg), investigators concluded that the decrease in arterial PO2 levels initiated events that led to acidosis, excess lactate formation, and intravascular sickling. They postulated that even though factors such as dehydration, increased blood viscosity, and hypercoagulability may have contributed to this syndrome, “the …. moderate-to-severe exercise after recent arrival at a relatively high altitude….” ultimately led to sickle crisis and sudden death. They also concluded that the variable hemoglobin S concentration (30–44%) among recruits spared some with sickle cell trait but not others.

Kark et al. [21] helped to establish the magnitude of the problem after a retrospective review of all the deaths among active-duty military between 1977 and 1981. The benchmark comprehensive review, written by Army Medical Corps physicians, concluded that the risk of sudden unexplained death in AA recruits with sickle cell trait was approximately 30-fold more than AA recruits without sickle cell trait and 40-fold more than that of non-AA recruits. Most deaths occurred during the first month of training and were associated with exertional activities requiring maximal effort. Most of the data implicated exertional heat illness, which had been somewhat arbitrarily subclassified into heat stroke, heat exhaustion, and heat injury. Deaths were attributed to exertional rhabdomyolysis if muscle necrosis were a major component of the clinical picture [22–24]. The degree of hyperthermia present during any of the syndromes was variable, and it was not always present during exertional rhabdomyolysis. Furthermore, in many cases, the true cause of death was difficult to determine; rhabdomyolysis may have been attributable to exercise, or mild muscle necrosis may have been augmented by hypotension during resuscitative measures and cardiac arrest.

Since it was strongly postulated that exertional heat illness was a likely trigger for the cascade of events, a 10-year (1982–1991) intervention study (unpublished-presented as abstract) [25] was designed to test this hypothesis. Army Drill sergeants monitored wet-bulb globe temperature and decreased exercise intensity with increased rest cycles and forced hydration as wet-bulb globe temperature rose. The gung ho culture and attitude of “fighting through it” was changed to one of early intervention at the first signs of struggle or duress. The study showed that these interventions eliminated or reduced the excess mortality associated with sickle cell trait in basic training. Others believe the preventive measures reduced the risk of death not by averting exercise—heat illness—but by making the exercises easier and ameliorating the forces favoring sickling [26]. Nevertheless, as a result of this study, in 1996, the Army ceased mandatory screening for the sickle gene.

A more recent study attempted to determine if the risk of exertional rhabdomyolysis and death varied among 47,944 AA soldiers who had undergone testing for HgbAS and were on active duty in the US Army between January 2011 and December 2014 [27]. The authors retrospectively reviewed the medical records of the soldiers and found that compared to those without HgbAS, sickle cell trait was not associated with a higher risk of death but was associated with a significantly higher risk of exertional rhabdomyolysis—even with the exertional injury universal precautions instituted since 2003 [28].

There are numerous published case reports of exertional sickling deaths in military recruits since 2003 [15–17]. Several factors may explain the higher incidence of sudden death in military recruits with HgbAS when compared with civilian athletes. The intensity of physical conditioning over 8–12 weeks is often implicated, and conditioning typically includes activities that raised the metabolic rate 10–14 times above the basal level. Other commonly cited factors include variable conditioning of recruits, viral syndromes, water deprivation in the field, and heavy protective military gear that may inhibit evaporation of sweat.

An age-associated increase in the death rate has been noted for those with sickle cell trait [24]. The death rate of a 28–29-year-old is eight-fold higher than that of a 17–18-year-old. The higher death rate may be attributed to the cumulative effects of renal papillary necrosis and resultant hyposthenuria [29]. Hyposthenuria is the only clinical condition consistently present in individuals with sickle cell trait. The condition tends to be intermittent and reversible initially but gradually may become structural, irreversible, and cumulative [30].

Each branch of the military has developed its own policy towards screening and prevention of exercise-related injury and sudden death. The Army does not routinely screen its recruits and relies on universal precautions [31]. The Air Force screens everyone and offers the option to decline service for each recruit positive for the sickle gene. If the recruit opts to stay in but endorses a history of symptoms associated with intravascular sickling or is found to have symptoms (attributable to intravascular sickling) during decompression at altitude, the individual is disqualified from hyperbaric duty, operational support flying, or intense physiological training [32]. The Navy screens recruits and identifies those with sickle cell trait by a neck tag and a red belt during strenuous exercise drills, and individuals are disqualified from special operations duty [33, 34]. The Marines screen all individuals and do not alter the routine of those with sickle cell trait, but as a department of the Navy, special operations duties are prohibited [34].

### Civilian Deaths

The vast majority of civilian deaths attributed to sickle cell trait has occurred in college athletes. The first recorded case was a 19-year-old football player from Coral Gables, Florida, who collapsed the first time during “conditioning” workouts in 1973 while at the University of Colorado in Boulder (5430 ft). The following year during another practice session in late August, he collapsed and later died [35]. His death was the NCAA’s first documented case of sudden death as a complication of sickle cell trait.

In collegiate football, between 1974 and 2010, there were 18 deaths related to sickling and reported in the lay press. There were 11 sickling-related deaths (0.08 per 100,000 participants) between 1999 and 2010 with 2 occurring in high school and 9 in college athletes. All 11 were AA and it was not known that 4 athletes had sickle cell trait until post-mortem testing [16]. All deaths occurred during sprinting or agility sessions during practice. Two deaths occurred during sprinting drills during the season’s first practice session.

In 2012, Harmon et al. [17] reviewed the causes of all cases of sudden deaths in NCAA student athletes from January 2004 through December 2008. There were 273 deaths and a total of 1,969,663 athlete-participant-years. Five deaths were associated with sickle cell trait and all occurred during physical exertion. All deaths associated with sickle cell trait occurred in AA Division 1 football players. The risk of exertional death in Division 1 football players with sickle cell trait was 1:827 which was 37 times higher than in athletes without sickle cell trait.

The timing and association of these deaths with maximal physical exertion during “conditioning” exercises have not been coincidental to the military experience. The NCAA attributes the deaths to an “intensity syndrome” not exertional heat illness as postulated by the Army. Ferster and Eichner [14] suggest that the inciting trigger for fatal sickling in Army recruits is sustained high-intensity physical exertion—without elevated ambient temperature or exercise heat illness.

Following the death of the University of Colorado football player, the NCAA in 1975 recommended testing all athletes for the sickle gene. The recommendation was rescinded in 1992 based on the absence of data that testing prevented death. Schools continued to test at highly variable rates. It was only after the family of a deceased student athlete with sickle cell trait sued the NCAA and stipulated mandatory sickle cell testing of all athletes as part of the settlement [36]. In April 2010, the NCAA adopted legislation that required screening of all athletes. This action required that incoming Division I athletes must be tested for HgbAS, show proof of a prior test, or sign a waiver releasing an institution from liability [37]. This went into effect in August 2010 for Division 1 schools and subsequent years for Division II and III schools [37, 38].

There are other civilian deaths associated with sickle cell trait. Most are case reports and range from firemen dying during training exercises [13], a 6-year-old playing in the park [13], a teenager running during forced participation in a juvenile justice boot camp [39], and a 13-year-old running from the police [40].

### Physical Findings

Military and civilian sickle cell trait-related deaths share many common features. The individuals were usually participating in a physical event that required sustained maximal exertion for at least a few minutes and typically complained of muscle weakness more than pain. Presumably, the collapse is due to generalized weakness unlike the abrupt collapse associated with cardiac dysrhythmias. The larger muscles (calves, quads, hamstrings, glutes) may look and feel normal as compared to the cramping associated with heat cramps. While typically alert and conscious, a cardinal feature is the increasing pain and weakness in the large working muscles.

Exertional sickling as a cause of sudden collapse has been under-recognized and often confused with heat stroke or cardiac dysrhythmias. Since it is very difficult for even the most seasoned clinician to differentiate between these syndromes, the first step (as in any clinical situation) is to get a history. Did the symptoms come on quickly—muscle cramping associated with heat illness usually has a prodrome of hours to minutes, and before cramping occurs, the athlete usually experiences some muscle twitching or twinges. Muscle pain or cramping due to exertional sickling usually comes on very quickly with no prodrome.

The muscle pain associated with heat illness/cramping is severe due to sustained full contraction of large muscle groups, especially the hamstrings and quads in football players, whereas in exertional sickling, the pain is usually milder. Prior to collapse, in exertional sickling, the athlete gradually weakens and the legs cannot support weight vs the sudden collapse of a cardiac dysrhythmia or the painful last few steps of a contracted large muscle. Many reports and analyses of multiple individuals support a “sickling syndrome” which quickly give way to rhabdomyolysis and associated hyperkalemia, hyperosmolality, acidosis, elevated creatine phosphokinase, and red cell dehydration. These are presumed to lead to intravascular sickling and microvascular occlusion culminating in exertional collapse [25, 26]. More recently, a summit of experts introduced new terminology, “exertional collapse associated with sickle cell trait (ECAST),” in an attempt to better systemize the clinical findings [38].

All vital signs and core temperature should be assessed and is generally > 104 °F (40 °C) to meet the definition of exertional heat illness. Alternative methods to measure body temperature (e.g., oral, tympanic, axillary, skin/forehead) are not accurate in a previously exercising athlete and should not be used [41, 42]. Exertional heat stroke is associated with central nervous system involvement as well as major organ and tissue dysfunction. Mental status change with this degree of hyperthermia is enough to label as exertional heat stroke and initiate rapid cooling.

We cannot overlook the kidney’s potential role in initiating and then propagating the cascade of events. It is well known that hemoglobin S is more likely to polymerize in acidemic, low oxygen tension, high osmolality environments which are all present in the renal medulla. This leads to morphologic changes of the vasa recta and well-described ischemia, microinfarctions, and papillary necrosis [43]. Painless hematuria (thought to be secondary to papillary necrosis) is well described, and one study of AA Veterans with HgbAS found a two-fold increased risk of hematuria [44]. Dehydration is a well-known feature of many of the reported cases of sudden death in sickle cell trait. In the presence of age-related hyposthenuria in nearly all individuals with sickle cell trait, is the kidney’s inability to maximally concentrate the urine the protagonist of this cascade or a surrogate marker of the underlying condition?

### Management

The NCAA and the military has taken the first steps in trying to identify at-risk individuals and targeted education for everyone involved. Individuals with sickle cell trait should be allowed to build endurance slowly and perform workouts at their own pace with frequent rest periods. Water must be consumed beyond the level of intake stimulated by thirst. Conditioning regimens should be slow and gradual, and older or obese individuals may need to take extra precautions or given less strenuous regimens. The workout routine and cycles should also be adjusted for ambient heat and altitude. Coaches and trainers should be counselled that if the sickle cell trait athlete show signs of struggling during any drill, the activity should be halted and medical attention sought immediately.

The first step is to lay the individual down and assess basic vital signs. Supplemental oxygen, if needed, and oral hydration should be given immediately if the individual can take by mouth. If core temperature is > 104 °F (40 °C) and heat stroke is suspected, then rapid cooling is required. In the event core temperature cannot be accurately assessed and heat stroke is a consideration, expert opinion suggests cooling until the patient begins to shiver or treating with cold water immersion for 15–20 min before attempting transport to a higher level of care [45]. If no immediate improvement is seen, then activation of Emergency Medical Services by calling 911 is warranted. While waiting for the ambulance, parenteral hydration with normal saline should be given if available and an automatic external defibrillator applied. The individual should be taken to the nearest Emergency Room as quickly as possible and the staff alerted to the possibility of fulminant rhabdomyolysis associated with sickle cell trait.

## Conclusions

All individuals participating in high-intensity organized sports should know their HgbS status. Many individual and environmental factors likely play a role in the series of events leading up to exertional collapse associated with sickle cell trait. These include sustained intense exercise, dehydration, elevated ambient temperatures, high altitude, fatigue, poor physical conditioning, and concurrent febrile illness. These factors lead to hyperviscosity which raises the intracellular concentration of HgbS and promotes intravascular sickling, occlusion of the microvasculature, and rhabdomyolysis.

There is now a need to focus on children and adolescents of middle and high school age. Although sickle cell screening of all newborn children is mandated in all 50 states, information about HgbAS status may be poorly communicated to affected individuals or their parents. Also, how do we manage the children who are not born in the USA and either who are not tested at birth or have no recollection of their sickle cell status and enlist to participate in team school sports?

All interested parties agree that those in the military, athletes, and coaches should receive education to reduce risks and formalize an adequate response if a situation does occur during rigorous physical exertion. Until conclusive physiologic and pathologic evidence are gathered, the reasonable assumption of a causal relationship between sickle cell trait, exertional collapse, and sudden death must be acknowledged so that consistent, prudent recommendations for our patients, athletes, the military, and the public can be developed.

## Abbreviations

AA: African American
ECAST: Exertional Collapse Associated with Sickle Cell Trait
EHI: Exercise Heat Illness
HBA, α2β2: Adult hemoglobin
HgbAS: Heterozygous sickle cell
HgbS: Sickle hemoglobin
HgbSS: Homozygous sickle cell
NCAA: National Collegiate Athletic Association
RYR1: Type I ryanodine receptors
